# *Helicobacter pylori* associated aberrant methylation genes in blood leukocyte and gastric mucosa

**DOI:** 10.7150/jca.64613

**Published:** 2021-10-28

**Authors:** Yang Zhang, Duo Chen, Lian Zhang, Jun-Ling Ma, Tong Zhou, Zhe-Xuan Li, Wei-Dong Liu, Wei-Cheng You, Kai-Feng Pan

**Affiliations:** 1 Key Laboratory of Carcinogenesis and Translational Research (Ministry of Education/Beijing), Department of Cancer Epidemiology, Peking University Cancer Hospital and Institute, Beijing, 100142, People's Republic of China.; 2 Linqu Public Health Bureau, Linqu, Shandong, 262600, People's Republic of China.

**Keywords:** Gastric cancer, *Helicobacter pylori*, Methylation biomarker, Blood leukocyte, Gastric mucosa

## Abstract

**Background and Aim:** Methylation alterations may be involved in *Helicobacter pylori*-associated gastric carcinogenesis. This study aims to explore the potential *H.pylori-*associated methylation biomarkers in blood leukocyte and gastric mucosa.

**Methods:** Five candidate *H.pylori*-associated aberrant methylation genes were selected from the previous genome-wide profiling panels and validated in blood leukocyte and gastric mucosa in multi-stages (case-control validation between *H.pylori* positive and negative subjects and self-control validation before and after anti-*H.pylori* treatment).

**Results:** *GNAS* methylation level was decreased in blood leukocyte (62.07% *v.s.* 46.33%, *p*<0.001) and gastric mucosa (56.30% *v.s.* 32.42%, *p*<0.001) of *H.pylori* positive subjects compared to negative controls. While, *MTERF1* methylation level was increased significantly in blood leukocyte (29.57% *v.s.* 56.02%, *p*<0.001) and gastric mucosa (31.10% *v.s.* 47.50%, *p*<0.001) of positive subjects compared to controls. After successful *H.pylori* eradication, the methylation levels were increased from 44.87% to 60.88% (*p*<0.001) for *GNAS* and decreased from 46.19% to 34.56% (*p*<0.001) for *MTERF1* in blood leukocyte. Similar increasing and decreasing methylation alterations were also found for the two genes after successful eradication in paired gastric mucosa. In TCGA database, an inverse relationship was found between *GNAS* methylation and mRNA expression (*r*=-0.12, *p*=0.027). The GC cases with higher *GNAS* expression levels showed significantly worse survival (HR, 2.09, 95%CI, 1.22-3.57,* p*=0.007) compared to lower expression subjects.

**Conclusions:** *GNAS* and *MTERF1* methylation levels may be affected by *H.pylori* infection in gastric mucosa and blood leukocyte. *GNAS* may be involved in advanced stage of GC development, although the possible mechanism still needs further study in precancerous lesions.

## Introduction

According to the cancer statistics from GLOBOCAN 2018, 2.2 million new cancer cases were attributable to carcinogenic infections. *Helicobacter pylori* (*H.pylori*) is the most important infectious agents and responsible for 810 000 new cancer cases. High burden of infection-attributable cancer driven by *H.pylori* was observed in eastern Asia (480 000 cases), especially in China (340 000 cases) 1.

Evidences suggest that *H.pylori*-induced chronic inflammation and epigenetic alterations may serve as one of the most important mechanisms in the process of gastric carcinogenesis 2. The degree of exposure to *H.pylori* infection may influence the accumulation of aberrant methylation in various genes, which produces an “epigenetic field of cancerization” in normal-appearing tissues. The severity of this field was found to be correlated with the predisposition to gastric cancer (GC) and recently was used to predict the risk of metachronous GC 3, 4.

Aberrant methylations in blood leukocyte DNA are considered as surrogate biomarkers for their easy accessibility and correlations with the risk of various cancers or endogenous and exogenous risk factors 5, 6. For example, global hypomethylation (*ALU*, *LINE1* and *Sat2*) 7, 8 and hypermethylation of specific genes (*IGFII* and *TUSC3*) 9, 10 were reported to be associated with GC or *H.pylori* infection. Our previous genome-wide profiling study has found a high correlation of methylation levels between blood leukocyte and matched gastric mucosa, which supports the methylation of blood leukocyte as a potential surrogate biomarker for the epigenetic alteration in gastric carcinogenesis. Furthermore, the panel of *H.pylori*-associated differentially methylated genes was preliminarily identified in blood leukocyte and in gastric tissue 11.

From the previous candidate *H.pylori*-associated aberrant methylation gene panel in blood leukocyte, our pilot validation found decreasing trends of* GNAS* and *LTBR* methylation levels after *H.pylori* eradication by denaturing high performance liquid chromatography (DHPLC) in a small sample size comparison 11. Besides *GNAS* and *LTBR*, *DKK3* was selected from the candidate panel according to literatures 11-13 for further validation in the present study. *MTERF1* and *HAUS5* were also selected from the candidate aberrant methylation panel overlapped between blood leukocyte and gastric tissue, in which the differentially methylated CpG sites were located both near CpG island and promoter regions 11.

Depending on the above selection strategy, the present multi-stage study validated the five candidate *H.pylori*-associated aberrant methylation genes by methylation specific real-time PCR in blood leukocyte as well as in gastric mucosa. We further evaluated the relationship between the potential non-invasive *H.pylori*-associated epigenetic biomarkers and precancerous gastric lesions. Possible clinical significance was also preliminarily investigated in GC using TCGA database.

## Methods

### Study design and population

Linqu county in Shandong province, China, is a high-risk area of GC 14. From 2011 to 2013, a large community-based randomized intervention trial to prevent GC was launched in Linqu county using ^13^C-urea breath test (^13^C-UBT) for *H.pylori* infection screening and a 10-day quadruple anti-*H.pylori* treatment for positive subjects 15. In 2012 and 2013, the National Upper Gastrointestinal Cancer Early Detection Project conducted endoscopic examinations and collected blood and fresh gastric biopsy samples in about 1500 Linqu county residents annually according to the inclusion criteria (aged 40-69, male or non-pregnant, non-lactating female, informed consent form signed). The exclusion criteria are as follows: blood clotting disorders, high blood pressure, liver disease, chronic obstructive pulmonary disease and inability to provide an informed consent.

For the current study, a total of 140 *H.pylori* positive subjects who were both treated in the intervention trial and received endoscopic examinations prior to and six months after treatment were enrolled in a two-step self-control validation. We identified the efficacy of intervention in the 140 subjects by a second ^13^C-UBT after treatment as successful and unsuccessful eradication regardless of what kind of therapies they were assigned. The two-step self-control validation includes a preliminary self-control comparison (18 successfully and eight unsuccessfully eradicated cases) and a secondary self-control comparison (66 successfully and 48 unsuccessfully eradicated cases). In addition, 47* H.pylori* negative and 47 positive subjects were randomly selected from the endoscopic examination participants with baseline *H.pylori* infection screening result in 2013 for case-control validation.

A written informed consent was obtained from each subject. The study was approved by Institutional Review Board of Peking University Cancer Hospital (2015KT67).

### Upper endoscopic examination and histopathology

The detailed procedure of endoscopy has been described elsewhere 16. Briefly, gastric mucosa was examined by experienced gastroenterologist using video endoscopes (Olympus). At least two biopsies were obtained from less curve of antrum, one for pathological diagnosis, one for DNA extraction and methylation detection. The gastric biopsy specimens were reviewed blindly by two pathologists according to the criteria proposed by the Chinese Association of Gastric Cancer 17 and Updated Sydney System 18. Each biopsy was diagnosed as normal, superficial gastritis (SG), chronic atrophic gastritis (CAG), intestinal metaplasia (IM) or dysplasia (DYS) based on the most severe histology.

### DNA preparation and methylation measurement

Blood leukocyte and gastric biopsy samples were frozen at -80 °C until DNA extraction. Genomic DNA was extracted from gastric biopsies using the QIAamp DNA Mini Kit (Qiagen, California, USA) and from blood leukocyte by standard proteinase K digestion and phenol-chloroform extraction. The extracted DNA was bisulfite-converted using EZ DNA methylation kit (Zymo Research) according to the manufacturer's instructions.

The methylation levels of the five candidate genes were detected by quantitative methylation-specific PCR using a StepOne Real-Time PCR System (Applied Biosystems) with the primers and probes as described in Table S1. The 20μl PCR reaction mixture contained 1×TaqMan Fast Universal PCR Master Mix (Applied Biosystems), 0.9 mmol/L each of forward and reverse primers, 0.25 mmol/L each of probes, and 20 ng of DNA at the following conditions: 95 °C for 20 seconds, followed by 40 cycles at 95 °C for one seconds, and 60 °C for 30 seconds. The efficiencies of all the genes PCR amplification were confirmed as nearly 100%. To normalize the input DNA, ACTB was used as the reference. The methylation levels of the candidate genes were calculated by dividing target gene/ACTB ratio of a sample by the target genes/ACTB ratio of positive control DNA treated by CpG methyltransferase (M.Sssl).

### *GNAS* and *MTERF1* methylation and mRNA expression in TCGA data

Because of the lack of GC cases and mRNA expression in our multi-stage validation, we accessed TCGA stomach cancer study data (http://www.xenabrowser.net) for the methylation and mRNA expression levels of *GNAS* and *MTERF1* in GC cases. A total of 11 CpG sites in the promoter region of *GNAS* (from cg19640589 to cg21625881) and 15 CpG sites in *MTERF1* (from cg10872641 to cg22709100) were selected from the Illumina HumanMethylation 450 DNA methylation data. The mRNA expression data were retrieved from Illumina HiSeq RNA-SeqV2 platform and log2 (RPKM+1) transformed. Other relevant clinical information was obtained including age, gender, pathological stage, *H.pylori* infection, targeted molecular therapy and radiation therapy.

### Statistical analysis

Wilcoxon Singed Ranks Test was used to compare the methylation levels before and after anti-*H.pylori* treatment. Mann-Whitney test was used to compare age and methylation levels between *H.pylori* positive and negative groups. We compared methylation levels among SG, CAG and IM/DYS groups by Kruskal-Wallis test. Odds ratios (ORs) with corresponding 95% confidence intervals (CI) were calculated by unconditional logistic regression to evaluate the relationships between methylation status and *H.pylori* infection or gastric lesion groups adjusting for age, sex, smoking, drinking and baseline pathological diagnosis. Spearman correlation analysis was performed to calculate the correlation coefficients (*r* values) between methylation levels in blood leukocyte and gastric mucosa, or between methylation and mRNA expression levels. The associations between methylation or expression with overall survival were assessed using hazard ratio (HR) and corresponding 95%CI by COX regression model adjusting for age, gender, pathological stage, *H.pylori* infection, targeted molecular therapy and radiation therapy.

## Results

### Characteristics of the subjects for multi-stage validation

For the two-step self-control validation, the distributions of age, gender, cigarette smoking, alcohol drinking and baseline pathology for the participants are shown in Table S2. No statistical differences were found between successful and unsuccessful treatment groups both in preliminary and secondary self-control validations (all *p*>0.05). For case-control validation, there are higher frequencies of advanced gastric lesions (CAG and IM/DYS) in *H.pylori* positive group compared with negative group (17.0%, 40.4% *v.s.* 8.5%, 12.8%, *p*=0.001), although no differences were found in age, gender, cigarette smoking and alcohol drinking (Table S3).

### Preliminary self-control validation for candidate aberrant methylation genes

For preliminary self-control validation, we detected *GNAS*, *LTBR* and* DKK3* methylation levels in blood leukocyte and *MTERF1, HAUS5* in both blood leukocyte and gastric tissue (Table 1). *GNAS* and *DKK3* methylation medians were increased in blood leukocyte after successful *H.pylori* eradication (*GNAS*: 46.42% *v.s.* 56.54%, *p*=0.035, *DKK3*: 25.68% *v.s.* 34.51%, *p*=0.035). No significant methylation alteration was found for *LTBR* in blood leukocyte before and after eradication (36.40% *v.s.* 43.92%, *p*=0.306). *MTERF1* methylation was markedly decreased by successful eradication both in blood leukocyte (56.79% *v.s.* 30.96%, *p*=0.001) and gastric mucosa (45.38% *v.s.* 36.84%, *p*=0.031). *HAUS5* methylation level was increased significantly in gastric mucosa (49.57% *v.s.* 60.84%, *p*=0.048). However, the increasing trend showed no statistical significance in blood leukocyte (35.74% *v.s.* 40.12%, *p*=0.528). All of the candidate genes showed no significant methylation changes before and after unsuccessful treatment (all *p*>0.05).

### Case-control validation for candidate aberrant methylation genes

The four significant genes in preliminary self-control validation were compared between *H.pylori* positive and negative cases (Table 2). *GNAS* showed marked methylation decreasing both in blood leukocyte (62.07% *v.s.* 46.33%, *p*<0.001) and gastric mucosa (56.30% *v.s.* 32.42%, *p*<0.001) in *H.pylori* positive subjects compared to negative ones. In addition, *MTERF1* methylation levels were increased significantly both in blood leukocyte (29.57% *v.s.* 56.02%, *p*<0.001) and gastric mucosa (31.10% *v.s.* 47.50%, *p*<0.001) of positive subjects compared to controls. No statistical differences of methylation levels were found for *DKK3* and *HAUS5* between *H.pylori* positive and negative groups (all *p*>0.05).

The correlations of the methylation levels between blood leukocyte and gastric mucosa were further analyzed in the 94 subjects. *MTERF1* methylation in blood leukocyte is correlated with that in gastric mucosa (*r*=0.50, *p*<0.001). Similar correlation can also be found for *GNAS* methylation levels in blood leukocyte and gastric mucosa (*r*=0.28, *p*=0.007). However, no significant correlations were found for *DKK3* and *HAUS5* methylation between the two kinds of samples (both *p*>0.05).

According to the methylation medians of *GNAS* (53.23%) and *MTERF1* (39.51%) in blood leukocyte of the total subjects, we used 50% and 40% as cut-off values and divided methylation levels into hyper- and hypo-methylation categories. Methylation status of* GNAS* was decreased (in blood leukocyte: OR, 0.16; 95%CI, 0.06-0.45, in gastric mucosa: OR, 0.09; 95%CI, 0.03-0.28) and *MTERF1* was increased (in blood leukocyte: OR, 25.96; 95%CI, 6.56-102.73, in gastric mucosa: OR, 8.70; 95%CI, 2.84-26.66) in *H.pylori* positive subjects compared to controls adjusting for age, sex, smoking, drinking and gastric pathology (Table 3).

### Secondary self-control validation for candidate aberrant methylation genes

We validated *GNAS* and *MTERF1* methylation in a secondary self-control comparison with larger sample size (Table 4). In 66 pairs of blood leukocyte samples,* GNAS* methylation median was increased from 44.87% to 60.88% (*p*<0.001) and *MTERF1* was decreased from 46.19% to 34.56% (*p*<0.001) by successful eradication. In the 37 pairs of gastric mucosa obtained from successful eradication group, similar increasing and decreasing methylation alterations were also found for the two genes (*GNAS*: 55.56% *v.s.* 69.47%, *p*=0.002;* MTERF1*: 35.95% *v.s.* 19.84%, *p*<0.001). No significant methylation alterations of the two genes were found in 48 pairs of blood leukocyte or 13 pairs of gastric mucosa samples collected from unsuccessful eradication group (all *p*>0.05).

### The associations between aberrant methylation genes and gastric lesions

We further investigated the relationship of the aberrant methylation genes with gastric lesions by combining the 94 case-control subjects and the 114 baseline subjects of the secondary self-control validation (Table 5). The total subjects consist of 103 SG, 74 CAG and 31 IM/DYS. In the blood leukocyte samples, *GNAS* methylation median showed decreasing trend with the severity of gastric lesions from 52.81% in SG to 46.20% in IM/DYS, *p*=0.053. In the 144 subjects possessing gastric mucosa samples, *GNAS* methylation median was also marginally decreased from 55.68% in SG to 37.87% in IM/DYS, *p*=0.053. While, *MTERF1* methylation levels showed no difference in various lesion groups (*p*=0.407 in blood leukocyte and 0.547 in gastric mucosa). Multivariate unconditional logistic regression found no significant relationship between advanced gastric lesions (CAG/IM/DYS) and *GNAS* or *MTERF1* methylation in blood leukocyte or gastric mucosa, all *p*>0.05 (Table S4).

### The correlation of methylation with mRNA expression and clinical relevance investigation in GC by TCGA database

We used TCGA-STAD database to investigate the correlation of methylation and mRNA expression in GC tissue. The average promoter methylation and mRNA expression levels of *GNAS* and *MTERF1* were retrieved from 372 gastric adenocarcinoma cases. Spearman correlation analysis revealed inverse relationships between promoter methylation and mRNA expression for *GNAS* (*r*=-0.12, *p*=0.027) and *MTERF1* (*r*=-0.55, *p*<0.001) in GC tissues.

**Figure 1:**
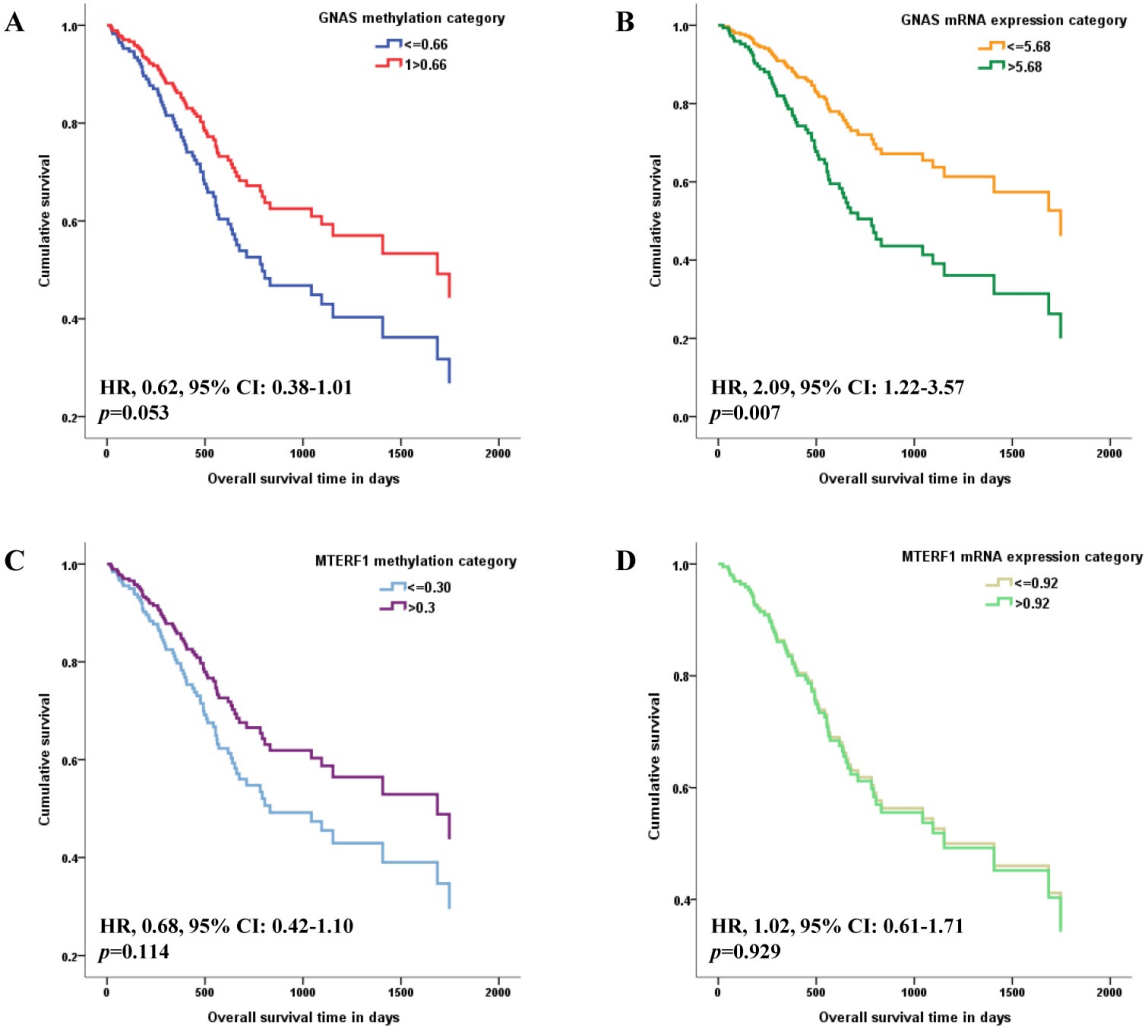
** Associations of candidate gene methylation and mRNA expression with overall survival of GC patients in TCGA database.** The medians of methylation or mRNA expression levels for *GNAS* and *MTERF1* in total subjects were used as cutoff values to divide GC cases into hyper- and hypo-methylation groups or higher and lower mRNA expression groups. Multivariate COX regression model found that overall survival is marginally better for GC subjects with hypermethylated *GNAS* promoter (**A**), and worse for GC subjects with higher *GNAS* mRNA expression levels (**B**) adjusting for age, gender, pathological stage, *H.pylori* infection, targeted molecular therapy and radiation therapy. However, no significant association was found between GC survival and *MTERF1* methylation (**C**) or mRNA expression status (**D**).

The methylation and mRNA expression levels of *GNAS* and *MTERF1* were further evaluated with GC survival in TCGA-STAD. Methylation or mRNA expression medians for* GNAS* and *MTERF1* in total subjects were used as cutoff values to divide GC cases into hyper- and hypo-methylation groups or higher and lower mRNA expression groups. Cox regression model found that the HR was 0.62 (95% CI: 0.38-1.01) for GC subjects with hypermethylated *GNAS* than hypomethylated subjects, although the *p* value showed no statistical significance (*p*=0.053, Figure 1A). In addition, the GC cases with higher *GNAS* mRNA expression levels showed significantly worse survival (HR, 2.09, 95% CI, 1.22-3.57,* p*=0.007) compared to lower expression subjects after adjusting for age, gender, pathological stage, *H.pylori* infection, targeted molecular therapy and radiation therapy (Figure 1B). No significant associations were found between GC survival and *MTERF1* methylation (HR, 0.68, 95% CI: 0.42-1.10, *p*=0.114, Figure 1C) or mRNA expression (HR, 1.02, 95% CI: 0.61-1.71, *p*=0.929, Figure 1D).

## Discussion

Depending on our previous genome-wide methylation profiles prior to and after anti-*H.pylori* treatment, the present study further validated that *GNAS* and *MTERF1* methylation levels in blood leukocyte and gastric mucosa are correlated, and may be affected by* H.pylori* infection. Especially, *GNAS* may also be involved in advanced stage of GC development.

In the preliminary self-control validation, the five candidate aberrant methylation genes were detected by quantitative methylation-specific PCR in 18 successful and eight unsuccessful* H.pylori* eradiation cases. Although the change of *LTBR* methylation shows no statistical significance, the methylation alterations of *DKK3*, *GNAS*, *MTERF1* and *HAUS5* are consistent with the previous methylation chip analysis 11, which suggests the high accuracy of our validation method.

*H.pylori* infection was previously reported to be associated with aberrant methylation in gastric mucosa or blood leukocyte DNA 19-21, however only few study evaluated the two kinds of samples at the same time. Our previous array analysis has compared the methylation levels between blood leukocyte and gastric mucosa in large-scale and found a high correlation 11, which suggests blood leukocyte methylation as a surrogate biomarker for gastric mucosa methylation. The present study further identified the decreasing *GNAS* and increasing *MTERF1* methylation levels in both blood leukocyte and gastric mucosa of *H.pylori* positive subjects compared to negative controls. The correlated methylation levels in the two tissues support *GNAS* and *MTERF1* methylation levels in blood leukocyte as potential surrogate biomarkers for *H.pylori*-associated methylation alterations in gastric mucosa. Chronic inflammation induced by *H.pylori* infection was reported to promote aberrant methylation in gastric mucosa 22, which may also be responsible for the correlated methylation alterations in blood leukocyte.

The relationship between *H.pylori* infection and methylation alterations can be further confirmed by anti-*H.pylori* intervention in addition to the case-control study. Some specific methylation changes (such as *CDH1, COX-2,* and *LOX*) were already reported in gastric mucosa after *H.pylori* eradication 23-25, which may be involved in gastric carcinogenesis. While no methylation alterations before and after eradication have been systemically evaluated both in gastric mucosa and in blood leukocyte. Our study identified that the hypomethylated *GNAS* and the hypermethylated* MTERF1* in *H.pylori* positive subjects may be reversed by successful eradication in the two kinds of samples. Furthermore, no significant methylation changes in unsuccessful treatment group may also help to suggest the methylation alterations of *GNAS* and *MTERF1* as the consequences of *H.pylori* infection.

The levels of aberrant methylation burden induced by *H.pylori* may increase with the period and density of the infection and finally accumulate in precancerous tissues producing an epigenetic field defect for cancerization 26. Our validation identified that aberrant *GNAS* and *MTERF1* methylation alterations can be induced by *H.pylori* infection. However, no independent relationships were found between *GNAS* or *MTERF1* methylation levels and advanced gastric lesions (such as CAG or IM/DYS) in the present study. Further confirmation in a larger number of precancerous lesion subjects is still needed.

Although no GC patients were included in our case-control and self-control validations, we accessed TCGA data and found inverse relationships between promoter methylation and mRNA expression for *GNAS* and *MTERF1* in GC tissues. Our result suggests that promoter methylation of *GNAS* and *MTERF1* may serve as one of the mechanisms for expression regulation, although the detailed functions still need further investigation. Worse overall GC survival was found to be associated with higher expression of *GNAS* in GC patients, which may imply an important role of *GNAS* expression activation in the advanced stage of GC development. The hypomethylation induced by *H.pylori* may serve as the epigenetic prerequisite and provide a potential alternation to promote *GNAS* expression in addition to the well-studied activating mutations via the Wnt/β-catenin pathway or the ERK1/2 MAPK pathway 27, 28. The correlated methylation alterations of *GNAS* in blood leukocyte and gastric mucosa may also suggest a potential non-invasive indicator for gastric epigenetic regulation, while the clinical significance still needs further validation.

The major strengths of our study lie in complementary design of combining case-control and self-control validations for *H.pylori*-associated differential methylation genes selected from the previous methylation chip screening. The significant *H.pylori*-associated methylation alterations were verified in blood leukocyte as well as in gastric mucosa, which may provide potential non-invasive indicator for gastric epigenetic regulation. A limitation in our study is the lack of GC subjects when we investigated the aberrant methylation status in various gastric lesions. However, TCGA database can provide us helpful information about gene methylation and expression in GC tissue to support our validation results.

For the five candidate methylation genes selected from our previous methylation chip screening, our case-control and self-control validations confirmed that *GNAS* and *MTERF1* methylation levels may be affected by *H.pylori* infection both in gastric mucosa and in blood leukocyte. Methylation of *GNAS* may serve as one of the mechanisms for expression regulation. *GNAS* may be involved in advanced stage of GC development, although the possible mechanism still needs further study in precancerous lesions.

## Supplementary Material

Supplementary tables.

## Figures and Tables

**Table 1:** Preliminary self-control validation for candidate aberrant methylation genes

Gene name	Methylation level medians (interquartile)% in successful eradication group (n=18)			Methylation level medians (interquartile)% in unsuccessful eradication group (n=8)		
	Before treatment	After treatment	*p*^†^ value	Before treatment	After treatment	*p*^†^ value
**In blood leukocyte**						
*GNAS*	46.42 (34.74-53.49)	56.54 (39.91-69.82)	0.035	49.80 (38.54-57.87)	57.88 (49.66-61.39)	0.161
*LTBR*	36.40 (23.83-50.43)	43.92 (24.01-67.00)	0.306	41.61 (30.10-55.65)	48.04 (35.39-59.25)	0.889
*DKK3*	25.68 (12.68-37.20)	34.51 (30.64-45.89)	0.035	31.60 (18.63-38.55)	19.97 (16.55-33.62)	0.161
**In gastric mucosa and blood leukocyte**						
***MTERF1***						
Blood leukocyte	56.79 (38.65-78.88)	30.96 (16.12-44.13)	0.001	62.90 (43.85-79.23)	42.91 (13.94-88.23)	0.401
Gastric mucosa	45.38 (36.28-61.15)	36.84 (30.32-55.53)	0.031	26.44 (23.00-52.78)	35.69 (30.15-40.23)	0.575
***HAUS5***						
Blood leukocyte	35.74 (24.06-62.05)	40.12 (33.51-57.28)	0.528	34.41 (22.65-58.73)	37.89 (21.43-45.49)	0.208
Gastric mucosa	49.57 (42.55-63.10)	60.84 (46.30-68.78)	0.048	31.64 (13.25-52.46)	41.78 (15.69-58.53)	0.484

^†^ Wilcoxon Singed Ranks Test, comparing the methylation level before and after anti-*H.pylori* treatment.

**Table 2:** Case-control validation for candidate aberrant methylation genes

Genes	Sample source	Methylation level median (quartile) %		*p* value^†^
		*H.pylori* negative group (n=47)	*H.pylori* positive group (n=47)	
*GNAS*	Blood leukocyte	62.07 (51.69-75.86)	46.33 (36.25-54.24)	<0.001
	Gastric mucosa	56.30 (42.96-66.55)	32.42 (25.87-42.81)	<0.001
*DKK3*	Blood leukocyte	32.22 (21.77-42.12)	24.36 (18.59-36.17)	0.076
	Gastric mucosa	34.81 (23.16-45.77)	29.31 (23.13-39.12)	0.354
*MTERF1*	Blood leukocyte	29.57 (18.64-37.74)	56.02 (39.91-80.62)	<0.001
	Gastric mucosa	31.10 (20.95-42.17)	47.50 (35.12-62.89)	<0.001
*HAUS5*	Blood leukocyte	57.91 (43.11-72.82)	56.43 (26.10-69.74)	0.255
	Gastric mucosa	49.06 (30.28-64.64)	46.17 (32.09-60.69)	0.443

^†^ Mann-Whitney test.

**Table 3:** Case-control validation for candidate gene methylation status

Genes	Sample source	Methylation status	*H.pylori* negative (n=47), N (%)	*H.pylori* positive (n=47), N (%)	OR (95% CI*)*^†^	*p* value ^†^
*GNAS*	Blood leukocyte	Hypomethylation (≤50.00%)	11 (23.4)	29 (61.7)	1.00	
		Hypermethylation (>50.00%)	36 (76.6)	18 (38.3)	0.16 (0.06-0.45)	0.001
	Gastric mucosa	Hypomethylation (≤50.00%)	14 (29.8)	39 (83.0)	1.00	
		Hypermethylation (>50.00%)	33 (70.2)	8 (17.0)	0.09 (0.03-0.28)	<0.001
*MTERF1*	Blood leukocyte	Hypomethylation (≤40.00%)	38 (80.9)	12 (25.5)	1.00	
		Hypermethylation (>40.00%)	9 (19.1)	35 (74.5)	25.96 (6.56-102.73)	<0.001
	Gastric mucosa	Hypomethylation (≤40.00%)	35 (74.5)	14 (29.8)	1.00	
		Hypermethylation (>40.00%)	12 (25.5)	33 (70.2)	8.70 (2.84-26.66)	<0.001

^†^ Unconditional logistic regression analysis adjusted for age, sex, smoking, drinking and gastric pathology.

**Table 4:** Secondary self-control validation for candidate gene methylation

	Methylation level medians (quartile)%					
	Successful eradication group			Unsuccessful eradication group		
	Before treatment (n=66)	After treatment (n=66)	*p*^†^ value	Before treatment (n=48)	After treatment (n=48)	*p*^†^ value
**Blood leukocyte**						
*GNAS*	44.87 (34.72-52.93)	60.88 (48.17-70.67)	<0.001	47.50 (35.28-64.81)	49.01 (39.80-65.93)	0.356
*MTERF1*	46.19 (34.62-69.71)	34.56 (19.79-50.84)	<0.001	34.62 (27.97-46.07)	40.68 (25.09-54.28)	0.351
**Gastric mucosa**						
*GNAS*	55.56 (39.74-69.45)	69.47 (59.76-77.07)	0.002	60.63 (58.40-77.88)	60.25 (47.32-66.26)	0.117
*MTERF1*	35.95 (25.27-52.74)	19.84 (15.61-31.81)	<0.001	41.73 (22.68-60.47)	31.84 (22.32-55.40)	0.753

^†^ Wilcoxon Singed Ranks Test, comparing the methylation level before and after anti-*H.pylori* treatment.

**Table 5:** Associations between candidate gene methylation and gastric lesions

Genes	Methylation level medians (quartile)%			*p* value^†^
	SG (n=103)	CAG (n=74)	IM/DYS (n=31)	
**Blood leukocyte**				
*GNAS*	52.81 (42.54-64.87)	46.12 (36.28-53.78)	46.20 (38.33-60.35)	0.053
*MTERF1*	37.40 (27.83-53.15)	42.34 (30.37-62.27)	42.09 (30.96-62.78)	0.407
**Gastric mucosa**				
*GNAS*	55.68 (38.62-65.36)	47.03 (32.42-62.03)	37.87 (26.07-56.37)	0.053
*MTERF1*	35.85 (25.43-55.39)	37.47 (23.93-49.88)	40.31 (30.62-57.15)	0.547

Abbreviations: CAG, chronic atrophic gastritis; DYS, dysplasia; IM, intestinal metaplasia; SG, superficial gastritis. ^†^ Kruskal Wallis test.

## Abbreviations

^13^C-UBT: ^13^C-urea breath test
CAG: chronic atrophic gastritis
CI: confidence interval
DYS: dysplasia
GC: gastric cancer
HR: hazard ratio
*H.pylori*: *Helicobacter pylori*
IM: intestinal metaplasia
OR: odds ratio
SG: superficial gastritis
