# Supplementary material for: Helicobacter pylori associated aberrant methylation genes in blood leukocyte and gastric mucosa
Source: J Cancer. 2021 Oct 28;12(24):7326–33. doi: 10.7150/jca.64613 (PMC8734399; doi:10.7150/jca.64613)
Supplement: Supplementary file 1 — Supplementary tables. [file jcav12p7326s1.pdf]

Table S1. Primer and probe for quantitative methylation-specific PCR detection

Gene name	Primer and probe	Sequence	Product (bp)
GNAS	Primer-F	5’-TTTATTTGGGGTTTCGGAGA-3’	146
	Primer-R	5’-TTCACGAACAACAACCCCTA-3’	
	Probe	6FAM-GGTTGTTTATGTTGACGGGAGTGTCGTC-BHQ	
LTBR	Primer-F	5’-TATCGTTTGGGGTTGCGAT-3’	199
	Primer-R	5’-TCTAAACTCGAACAACCGCC-3’	
	Probe	6FAM-TAGGAGTATGGCGGTTATTCGGTTAGGC-BHQ	
DKK3	Primer-F	5’-AGGGGTTAGGGTTTGAATCG-3’	146
	Primer-R	5’-CCCCATCCTCGAACACAA-3’	
	Probe	6FAM-TCGGTTGTTTTGGCGAGGGTTTTAGTAT-BHQ	
HAUS5	Primer-F	5’-TTAGGAGGTTGAGGTAGTAGAATCG-3’	188
	Primer-R	5’-CTATTCCACTTCAAATCGTAAAACA-3’	
	Probe	6FAM-AGATTGCGTTGAGTCGAGATcgGGTTAT-BHQ	
MTERF1	Primer-F	5’-CGACGATACGTGGTGGAAA-3’	134
	Primer-R	5’-ACCCGCTTATCCTACACCAA-3’	
	Probe	6FAM-TAACGCGATGTCGATGAATATTTCGGC-BHQ	

Table S2 Characteristics of the subjects for preliminary and secondary self-control validations

Characteristics		Successfully eradicated group	Unsuccessfully eradicated group	*p* value
Preliminary self-control validation		N=18	N=8	
Age	Median (interquartile)	48.0 (45.8-52.3)	50.0 (43.5-54.0)	0.676 †
Gender	Male	10 (55.6%)	7 (87.5%)	0.190 ‡
	Female	8 (44.4%)	1 (12.5%)	
Smoking	Yes	6 (33.3%)	2 (25.0%)	>0.999 ‡
	No	12 (66.7%)	6 (75.0%)	
Alcohol intake	Yes	10 (55.6%)	6 (75.0%)	0.420 ‡
	No	8 (44.4%)	2 (25.0%)	
Baseline pathology	SG	8 (44.4%)	4 (50.0%)	0.950 ‡
	CAG	7 (38.9%)	3 (37.5%)	
	IM/DYS	3 (16.7%)	1 (12.5%)	
Secondary self-control validation		N=66	N=48	
Age	Median (interquartile)	51.5 (45.0-57.0)	48.5 (45.0-52.8)	0.060 †
Gender	Male	35 (53.0%)	29 (60.4%)	0.433 §
	Female	31 (47.0%)	19 (39.6%)	
Smoking	Yes	23 (34.8%)	20 (41.7%)	0.458 §
	No	43 (65.2%)	28 (58.3%)	
Alcohol intake	Yes	24 (36.4%)	17 (35.4%)	0.917 §
	No	42 (63.6%)	31 (64.6%)	
Baseline pathology	SG	26 (39.4%)	20 (41.7%)	0.966 §
	CAG	29 (43.9%)	20 (41.7%)	
	IM/DYS	11 (16.7%)	8 (16.7%)	

Abbreviations: CAG, chronic atrophic gastritis; DYS, dysplasia; IM, intestinal metaplasia; SG, superficial gastritis.

†Mann-Whitney test

‡Fisher's Exact test

§χ^2Test

Table S3 Characteristics of the subjects for case-control validation

Characteristics		*H. pylori* (-) N=47	*H. pylori* (+) N=47	*P* value
Age	Median (interquartile)	50.0 (45.0-54.0)	49.0 (45.0-54.0)	0.823 †
Gender	Male	29 (61.7%)	27 (57.4%)	0.674 ‡
	Female	18 (38.3%)	20 (42.6%)	
Smoking	Yes	14 (29.8%)	13 (27.7%)	0.820 ‡
	No	33 (70.2%)	34 (72.3%)	
Alcohol intake	Yes	20 (42.6%)	22 (46.8%)	0.678 ‡
	No	27 (57.4%)	25 (53.2%)	
Pathology	SG	37 (78.7%)	20 (42.6%)	0.001 §
	CAG	6 (12.8%)	19 (40.4%)	
	IM/DYS	4 (8.5%)	8 (17.0%)	

Abbreviations: CAG, chronic atrophic gastritis; DYS, dysplasia; IM, intestinal metaplasia; SG, superficial gastritis.

† Mann-Whitney test

‡ Pearson's Chi-square test

§ Fisher's Exact test

Table S4 *GNAS* and *MTERF1* methylation status and gastric lesions

Genes	Sample source	Methylation status	SG N (%)	CAG/IM/DYS N (%)	OR (95% CI) †	*p* value †
Blood leukocyte	*GNAS*	Hypomethylation (≤50.00%)	49 (47.6)	64 (61.0)	1	
		Hypermethylation (>50.00%)	54 (52.4)	41 (39.0)	0.93 (0.50-1.72)	0.809
	MTERF1	Hypomethylation (≤40.00%)	56 (54.4)	49 (46.7)	1	
		Hypermethylation (>40.00%)	47 (45.6)	56 (53.3)	0.93 (0.50-1.73)	0.822
Gastric mucosa	*GNAS*	Hypomethylation (≤50.00%)	31 (39.7)	38 (57.6)	1	
		Hypermethylation (>50.00%)	47 (60.3)	28 (42.4)	0.58 (0.28-1.22)	0.151
	MTERF1	Hypomethylation (≤40.00%)	43 (55.1)	36 (54.5)	1	
		Hypermethylation (>40.00%)	35 (44.9)	30 (45.5)	0.81 (0.37-1.78)	0.597

Abbreviations: CAG, chronic atrophic gastritis; DYS, dysplasia; IM, intestinal metaplasia; SG, superficial gastritis.

† Unconditional logistic regression analysis, adjusted for age, sex, smoking, drinking and *H. pylori* infection status
